# P-595. Epidemiological characteristics and molecular investigations for human-bite ticks in Japanese spotted fever endemic areas

**DOI:** 10.1093/ofid/ofaf695.809

**Published:** 2026-01-11

**Authors:** Shinnosuke Fukushima, Hidemasa Akazawa, Hideharu Hagiya

**Affiliations:** Department of Bacteriology, Okayama University Graduate School of Medicine, Dentistry and Pharmaceutical Sciences, Okayama, Okayama, Japan; Okayama University Hospital, Okayama City, Okayama, Japan; Okayama University Hospital, Okayama City, Okayama, Japan

## Abstract

**Background:**

Tick-borne diseases (TBDs) have been a concern worldwide, as tick habitats and active periods are expanding due to global warming. Among TBDs, Japanese spotted fever (JSF) is the most dominant in Western Japan. However, the epidemiology of the tick species that cause tick bites or the pathogens they carry is lacking. To clarify the pathogenic microorganisms carried by ticks that bite humans and the risk of developing tick-borne infections in JSF endemic areas.

**Figure:**
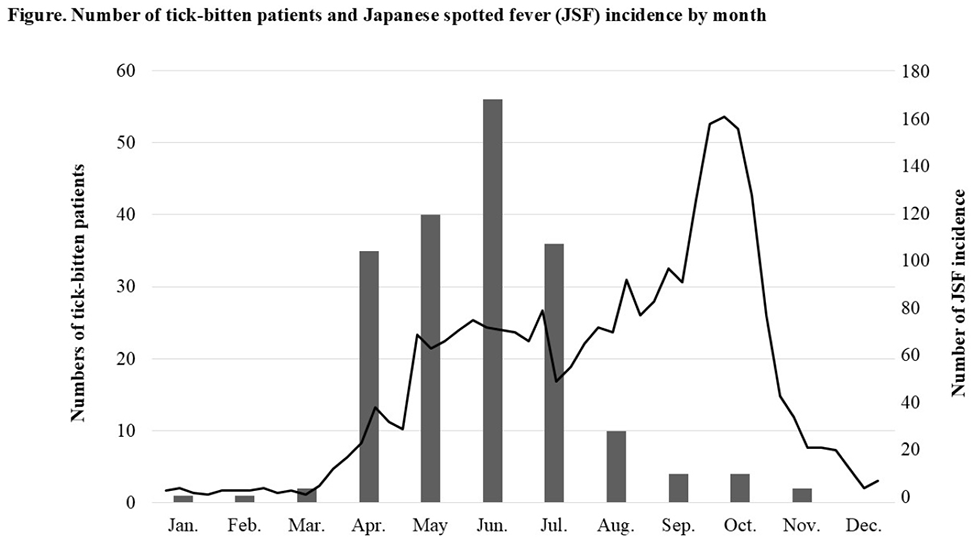
Number of tick-bitten patients and Japanese spotted fever (JSF) incidence by month

Tick-bitten patients were most prevalent in June in this study, and the incidence of JSF in Japan was highest in October between 2016 and 2022, based on the Infectious Diseases Weekly Report.

**Methods:**

A total of 10 medical institutions in Hiroshima, Okayama, and Kagawa prefectures in Japan were prospectively examined from May 2023 to December 2024 for the collection of tick specimens, which were found in patients presenting with tick bites. The estimated location of the bite, date, patient age, tick species identification, and the pathogens of TBDs were evaluated.

**Results:**

During the 20-month study period, a total of 191 ticks were collected from 181 patients, with a high proportion of women and those aged 60 years or older (30.9%). The seasonal distribution demonstrated peak incidence during April to July, with maximum prevalence observed in June (29.3%). *Amblyomma testicularis* constituted the predominant species, accounting for 152 (79.6%), followed by *Haemaphysalis hystrics* (7.3%) and *H. longicornis* (6.8%). Regarding developmental stages, nymphs represented the majority (83.8%), while adults and larvae comprised 14.6% and 1.0%, respectively. Twenty-eight tick species were found to carry *Rickettsia* species, including three distinct species: *Rickettsia tamurae*, *R. monacensis*, and uncultured *Rickettsia* species. However, molecular analysis failed to detect *Ehrlichia* spp. and *Anaplasma* spp., *Francisella tularensis*, and *R. japonica*. Throughout the year, the incidence of JSF rises in April, both nationwide and in JSF endemic areas, and reaches its peak in October.

**Conclusion:**

An epidemiological incongruity was observed between the incidence of tick bites and notified JSF cases, with molecular surveillance failing to detect *R. japonica*-harboring ticks among the collected specimens. Further epidemiological data regarding tick-borne pathogen prevalence will help constitute valuable surveillance information with significant clinical utility for disease diagnosis and management.

**Disclosures:**

All Authors: No reported disclosures

